# Comparing ^68^Ga-Pentixafor,^18^F-FDG PET/CT and Chemokine Receptor 4 Immunohistochemistry Staining in Breast Cancer: A Prospective Cross Sectional Study †

**DOI:** 10.3390/cancers17050763

**Published:** 2025-02-24

**Authors:** Bawinile Hadebe, Lerwine Harry, Lerato Gabela, Thembelihle Nxasana, Nontobeko Ndlovu, Venesen Pillay, Siphelele Masikane, Maryam Patel, Dineo Mpanya, Ines Buccimaza, Mpumelelo Msimang, Colleen Aldous, Mike Sathekge, Mariza Vorster

**Affiliations:** 1 Department of Nuclear Medicine, College of Health Sciences, University of KwaZulu Natal, Private Bag X54001, Durban 4001, South Africa; 2 Inkosi Albert Luthuli Central Hospital, Durban 4001, South Africa; 3 Department of Nuclear Medicine, School of Medicine, Faculty of Health Sciences, Sefako Makgatho Health Sciences University, Pretoria 0208, South Africa; 4 Department of Surgery, University of KwaZulu Natal, Durban 4001, South Africa; 5 Department of Anatomical Pathology, National Health Laboratory Service, Durban 4001, South Africa; 6 Department of Genetics, College of Health Sciences, University of KwaZulu Natal, Durban 4001, South Africa; 7 Department of Nuclear Medicine, Faculty of Health Sciences, University of Pretoria, Pretoria 0002, South Africa

**Keywords:** CXCR4, breast cancer, ^68^Ga-Pentixafor

## Abstract

*Background*. CXCR4 is a chemokine receptor that is frequently overexpressed in invasive breast cancer and plays a major role in tumor proliferation, aggressiveness and metastasis. The aim of this prospective study was to establish the value of CXCR4-directed PET imaging in patients with breast cancer using the novel CXCR4-targeted PET probe ^68^Ga-Pentixafor by comparing it with ^18^F-FDG PET/CT (*n* = 40). *Materials and methods*. In this prospective cross-sectional study, fifty-one patients with breast cancer aged 36–81 (median (Q1-Q3) 51 (42.5–63)), *n* = 47 (92%) with initially diagnosed and *n* = 4 (8%) patients with recurrent breast cancer, underwent CXCR4-targeted PET imaging using ^68^Ga-Pentixafor. Maximum standardized uptake values (SUVmax), total lesion glycolysis (TLG) or total lesion uptake (TLU), metabolic tumor volume (MTV) and tumor-to-background ratios (TBR) of tumor lesions were measured and correlated with pathological prognostic factors, molecular subtypes and CXCR4 immunohistochemistry (IHC) staining. ^18^F-FDG PET/CT images were available in 40 of 51 cases (82%) and were compared semi-quantitatively. The patients were followed up for a median of 11 months (range 4–80 months) to determine whether CXCR4 expression correlated with survival. *Results*. ^68^Ga-Pentixafor-PET/CT was visually positive in 49/51 (96%) of the cases; in addition, [^18^F]FDG demonstrated a higher SUVmax compared to ^68^Ga-Pentixafor. The mean SUVmax was 7.26 ± 2.84 and 18.8 ± 9.1 for ^68^Ga-Pentixafor and [^18^F]FDG, respectively. Thirty-seven percent (18/51) of patients had triple-negative breast cancer and 25/51 (49%) had estrogen receptor (ER+) disease. There was a statistically significant correlation between tumor grade, proliferative index (Ki-67) and SUVmax obtained from ^68^Ga-Pentixafor PET *p* = 0.002. There was no correlation between the SUVmax obtained from ^68^Ga-Pentixafor and PET molecular subtypes, estrogen receptor (ER), progesterone receptor (PR) or human epidermal growth factor receptor 2 (HER2) status; however, triple-negative breast cancers had more avid ^68^Ga-Pentixafor accumulation compared to luminals A and B. The median (Q1–Q3) ^68^Ga-Pentixafor TLU was significantly higher in HIV-positive (376 (219–881)) compared to HIV-negative (174 (105–557)) breast cancer patients. *Conclusions*. In conclusion, ^68^Ga-Pentixafor had a sensitivity of 96% and a specificity of 100% for detecting primary breast cancer; in addition, ^68^Ga-Pentixafor exhibited significantly higher uptake in patients with higher tumor grade, high proliferative index and triple-negative breast cancer (TNBC), as well as HIV-infected breast cancer patients, highlighting the potential clinical utility and prognostic role of CXCR4-targeted PET imaging in aggressive breast cancer. Notably, ^68^Ga-Pentixafor complements ^18^F-FDG by detecting more metastasis in the brain and the skull where FDG has limitations, while ^18^F-FDG remains superior for detecting skeletal metastasis. Future research should further explore the potential of CXCR4-targeted PET imaging in selecting patients with triple-negative breast cancer and high-grade breast cancer who may benefit from CXCR4-targeted therapies, particularly in the context of HIV co-infection.

## Simple Summary

This prospective cross-sectional study explored the complementary role of ^68^Ga-Pentixafor in ^18^F-FDG in patients with breast cancer using PET/CT imaging. ^68^Ga-Pentixafor-PET/CT was visually positive in 49/51 (96%) of the cases; however, ^18^F-FDG demonstrated higher tracer accumulation compared to ^68^Ga-Pentixafor. Indicators of prognosis in breast cancer, such as the proliferative index (Ki67) and Bloom Richardson grade, were correlated with a higher uptake (SUVmax) of ^68^Ga-Pentixafor. Also, HIV-infected patients with breast cancer and patients with triple-negative breast cancer had a higher accumulation of ^68^Ga-Pentixafor in primary tumors. Lastly, ^68^Ga-Pentixafor detected more brain and skull metastasis compared to ^18^F-FDG, whereas ^18^F-FDG detected more bone marrow metastasis. Therefore ^68^Ga-Pentixafor cannot replace ^18^F-FDG in the detection of breast cancer but plays a complementary role by detecting lesions in sites where ^18^F-FDG is limited by physiological tracer accumulation. ^68^Ga-Pentixafor has a role in the prognostication of patients and selecting potential candidates for therapies targeting CXCR4, particularly in the setting of HIV co-infection.

## 1. Introduction

Breast cancer is the most common malignancy and leading cause of cancer-related death in women, accounting for one in four cancer cases in women globally [1]. In Africa, breast cancer is the leading cause of cancer-related morbidity and mortality in females, with a reported 2,308,897 new cases and 665,684 deaths in 2024 [2]. Invasive breast cancer includes a heterogeneous group of tumors with a wide range of histologic subtypes, treatment response and prognosis [3]. Early diagnosis and appropriate treatment selection are crucial for improved survival; however, in our setting, patients often present with locally advanced or even metastatic disease, which is more challenging to treat [4]. In addition, triple-negative breast cancer, which is more aggressive and lacks targeted therapy, is more common in the African population [5]. Also, hormone-responsive and HER2+ breast cancers often progress or become unresponsive to targeted therapies; therefore, there is a need for more targeted therapies for breast cancer [6].

Currently, the highest diagnostic accuracy in the diagnosis of primary breast cancer and metastases is provided by ultrasonography, mammography, dedicated breast MRI and ^18^F-FDG PET/CT [5]. ^18^F-FDG PET/CT is indicated in patients with locally advanced breast cancer, high-grade molecular subtypes, triple-negative breast cancer or when conventional imaging modalities are equivocal [7]. ^18^F-FDG has a lower sensitivity in the detection of lobular cancer, as well as luminal A molecular subtypes, and it requires patient preparation, which is inconvenient and limits the scheduling of patients. In the recent past, a growing need for novel molecularly targeted approaches has emerged, enabling high-specificity diagnostics as well as personalized therapies with the appropriate molecular target due to the high toxicity of chemotherapy agents and the high incidence of resistance to chemoradiation [6,8].

Prognostic markers are crucial for optimizing treatment strategies and improving overall outcomes in breast cancer patients. They help identify patients with more aggressive diseases who require more frequent follow-ups due to a higher likelihood of recurrence and metastasis [9]. Hormone therapies, such as selective estrogen receptor modulators, estrogenic receptor degraders, aromatase inhibitors and luteinizing hormone-releasing hormone (LHRH) agonists, have contributed significantly to decreasing breast cancer mortality [10]. However, only 70% of breast cancers express the estrogen receptor (ER), and therefore, chemotherapy and therapies targeting genes with oncogenic alterations and related signaling pathways such as human epidermal growth factor receptor 2 (HER2), epidermal growth factor receptor (EGFR), BRAF, MAP2K1, ALK, CXCR4, etc., have been developed, based on advances in the understanding of molecular cancer biology.

Chemokine receptor 4 (CXCR4), the receptor of chemokine CXCL12/SDF-1 (stromal cell-derived factor-1) has been identified as a potential target for breast cancer imaging and therapy [11]. CXCR4 is expressed by cancer cells and inflammatory cells in the tumor microenvironment (TME) [12]. The binding of CXCR4 to its ligand CXCL12 activates the CXCL12/CXCR4 axis, which regulates tumor angiogenesis, promotes tumor metastasis, mediates immune dysfunction and plays a pivotal role in downstream cell-proliferation, migration and drug resistance [13].

CXCR4 expression promotes breast cancer metastasis to organs where there is an abundance of its ligand, SDF-1/CXCL12, such as the lymph nodes, brain, skeleton/bone marrow, liver and lungs [13]. The CXCL12 concentration gradient then drives the extravasation of CXCR4-positive tumor cells in circulation, leading to organ-specific metastasis [12]. Thus, tumors demonstrating a high expression of CXCR4 on IHC staining are associated with more extensive metastasis to lymph nodes compared to those with low levels of CXCR4 [14]. Also, CXCR4 expression was shown to be higher in metastatic sites as compared to primary tumors [15]. CXCR4 overexpression is associated with significantly reduced disease-free survival (DFS) and overall survival (OS) [12].

^68^Ga-Pentixafor is a novel radio-labeled CXCR4 ligand for PET/CT imaging. [16]. Its feasibility in breast cancer was shown in a retrospective analysis by Vag et al. in 18 patients with breast cancer where a comparison between [^68^Ga]-Pentixafor PET/CT and ^18^F-FDG PET/CT was performed in 8/18 patients [12]. The standardized uptake value (SUVmax) obtained during [^18^F]-FDG PET was higher than in CXCR4-targeted PET in all cases. However, it has not yet been established whether CXCR4 is a potential target for imaging and therapy in breast cancer.

Several CXCR4 antagonists have been studied in breast cancer models with encouraging outcomes [17,18]. They enhance the chemosensitivity to several chemotherapeutic medicines, such as docetaxel, potentially enhancing their antitumor activity [19]. These CXCR4 antagonists have potential as anticancer drugs for the treatment of breast cancer because they prevent the CXCR4 receptor from attaching to its ligand, SDF-1 [20], and therefore suppress tumor proliferation and metastasis.

With this background in mind, this study aimed to assess the diagnostic and prognostic utility of ^68^Ga-Pentixafor in the assessment of breast cancer. The objectives of this study were to (1) perform a comparative analysis of ^68^Ga-Pentixafor PET/CT and ¹⁸F-FDG PET/CT in terms of tracer uptake and lesion detectability, (2) determine the association between ^68^Ga-Pentixafor PET/CT uptake and the histopathologic and molecular prognostic markers of breast cancer, (3) investigate differences in ^68^Ga-Pentixafor uptake between HIV-positive and HIV-negative breast cancer patients, (4) assess the potential of ^68^Ga-Pentixafor PET/CT as a prognostic biomarker for predicting survival and treatment response in breast cancer and (5) correlate PET/CT findings with CXCR4 expression determined through immunohistochemistry (IHC) staining.

## 2. Methods

### 2.1. Patients

**Figure 1:**
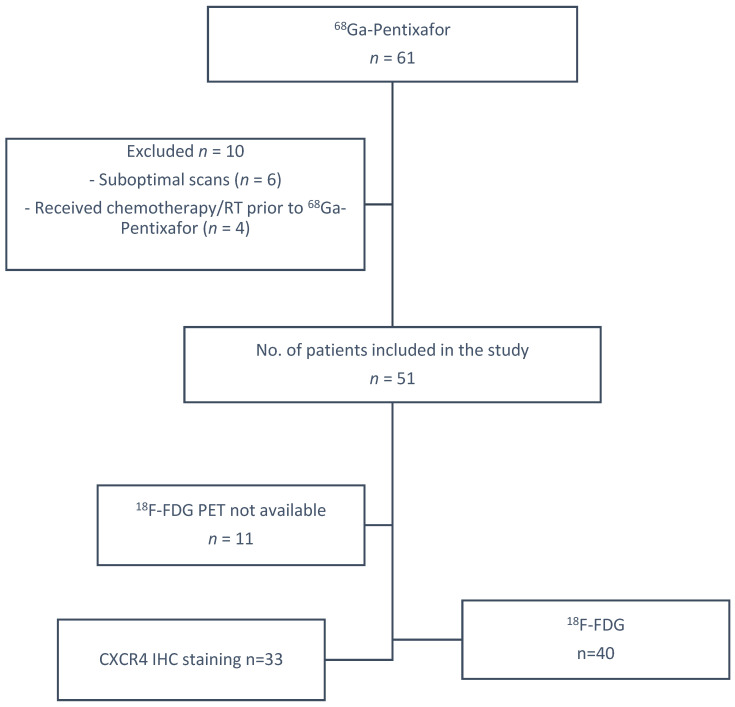
Summary of patient selection parameters.

This prospective cross-sectional study included 51 patients (median age (Q1–Q3) (42.5–63)) with histologically confirmed primary breast cancer, examined between June 2022 and August 2023. Part of this cohort has been described previously [21]. Inclusion criteria were age ≥18 years, biopsy-proven primary diagnosed or recurrent breast cancer and informed consent. Exclusion criteria were technically suboptimal scans and synchronous malignancy. Among the cohort, four of the patients had recurrent breast cancer following treatment. All patients underwent ^68^Ga-Pentixafor PET/CT. Forty patients additionally received a diagnostic ^18^F-FDG PET/CT for staging purposes within 1 month of ^68^Ga-Pentixafor imaging, as shown in Figure 1. No therapeutic interventions were administered between the two imaging modalities.

Informed consent was obtained from the patients for the scan and to access their hospital records. This study was approved by the Human Research Ethics Committee of the University of KwaZulu Natal (protocol reference number: BREC/00003636/2021). All procedures were performed in accordance with the ethical standards of the institutional research committee in alignment with the 1964 Helsinki Declaration and its later amendments.

### 2.2. Radiochemistry of ^68^Ga-Pentixafor

The synthesis of ^68^Ga-Pentixafor was performed in a semi-automated, GMP-compliant procedure using a GRP^®^ module (SCINTOMICS GmbH, Gräfelfing, Germany) equipped with a disposable single-use cassette kit (ABX, Redeberg, Germany). The eluate (^68^Ga^3+^ in 0.6 M HCl) of a ^68^Ge/^68^Ga-generator (Isotopen Technologies, Garching, Germany) was transferred to a cation exchange cartridge, eluted with 5 mL NaCl, added to a solution of 40 µg Pentixafor (PentixaPharm AG, Berlin, Germany) in HEPES-buffer and heated for 6 min at 105 °C. The product was immobilized on a SepPak C18 cartridge, washed with water and eluted with ethanol/water 50/50. The eluate was passed through a sterile filter (0.22 µm) into a sterile vial and diluted with phosphate buffer solution to a total volume of 15 mL. Radiochemical purity was determined by thin-layer chromatography using 0.1 M ammonium acetate. Radiochemical purity of the prepared derivatives was found to be >95% for all the derivatives. The median specific activity was 3.3 MBq/µg for ^68^Ga-Pentixafor.

### 2.3. ^68^Ga-Pentixafor PET/CT Imaging Procedure

There was no specific patient preparation for the ^68^Ga-Pentixafor PET/CT scan. The injected activity of ^68^Ga-Pentixafor was (1.4–4 MBq/kg) and ranged between 78 and 210 MBq per patient. We obtained whole-body (vertex to mid-thigh) PET/CT images at 60 min post-tracer injection. PET imaging was acquired in 3D mode at 7 min per bed position. We used CT data for attenuation correction and the anatomic delineation of lesions. We performed image reconstruction using TruX+ TOF (ultraHD-PET) (two iterations, 21 subsets) followed by post-reconstruction filtering with a Gaussian filter applied at 5.0 mm FWHM.

### 2.4. [^18^F]FDG PET/CT Imaging Procedure

Patient preparation for [^18^F]FDG PET/CT included a minimum of 4 hr of fasting as per the published guidelines. Blood glucose before [^18^F]FDG injection was less than 7.1 mmol/L in all cases. The injected activity of [^18^F]FDG was between 2 and 4 MBq/kg. We imaged patients at approximately 60 min post-injection. All patients were imaged on a Biograph 64 mCT slice PET/CT scanner (Siemens Medical Solutions, Lincolnshire, IL, USA). A vertex to mid-thigh CT scan was performed with parameters adjusted for patients’ weight (120 KeV, 40–150 mAs) with a section width of 5 mm and pitch of 0.8. The unenhanced CT was used for attenuation correction and anatomical localization.

### 2.5. Image Analysis

The ^68^Ga-Pentixafor PET/CT and ^18^F-FDG PET/CT images were reviewed by three Nuclear Medicine Physicians with experience reporting PET/CT images who were blinded to the clinical data and pathological findings. Reconstructed images were displayed as maximum intensity projection images, PET, CT and fused PET/CT in the axial, coronal and sagittal planes on a dedicated workstation equipped with syngo via software version 08.12 (Siemens Medical Solutions, Lincolnshire, IL, USA). The lesions were counted in each study and correlated with histopathology where available; when histology was not available (for suspected metastasis), conventional imaging and clinical follow-up were used to differentiate benign from malignant lesions.

### 2.6. Semi-Quantitative Analysis

For both tracers, images were analyzed to evaluate tracer accumulation in the primary tumor, nodal and distant metastases. Nodal metastases were differentiated from inflammation based on the distribution of tracer uptake and morphological features observed on the unenhanced CT. Any disagreements between the three readers were resolved through consensus.

Semi-quantitative analyses were conducted by delineating a volume of interest (VOI) over target lesions using an isocontour threshold of 40% for both tracers. Manual segmentation of the lesion was performed. The following parameters were measured and correlated with pathological prognostic factors and molecular subtypes: SUVmax, total lesion glycolysis (TLG) or total lesion uptake (TLU), metabolic tumor volume (MTV) and tumor-to-background (T/B) ratios of tumor lesions. TLG or TLU was calculated as the product of the VOI average SUV (SUVmean) multiplied by the corresponding MTV. Metabolic tumor volume (MTV) was defined as the volume of tissue demonstrating increased FDG or Pentixafor uptake on PET imaging. For TBR calculations, the SUVmax of the primary lesion was divided by the SUVmean of the normal liver or breast background tissue. For the TBR(liver), a 3 cm^3^ volume of interest (VOI) was placed on the right lobe of the liver and the corresponding SUVmean was recorded as the background for the liver. For the TBR(breast), a 2 cm^3^ VOI was placed on the contralateral normal breast tissue in the same quadrant as the lesion, and the SUVmean from this region served as the breast background reference.

### 2.7. Pathological Analysis

Histology of primary breast lesions was obtained by image-guided biopsy in all cases. The tissues were fixed in 10% neutral buffered formalin, dehydrated and cut into 2 μm-thick sections. Tumor classification according to the WHO 2012 criteria and assessment of the tumor biology (estrogen receptor, ER; progesterone receptor, PR; human epidermal growth factors receptor 2, HER2) was performed in routine pathological diagnostics. The proliferation index was assessed by immunohistochemistry using Ki67. In accordance with the St. Gallen guidelines, we used a local Ki67 cut-off value of 25% to differentiate between luminal A and luminal B tumors [22]. Tumors were categorized as follows: luminal A (ER+ and/or PR+, HER2/neu− and Ki67 < 25%), luminal B HER2− (ER+ and/or PR+, HER2/neu− and Ki67 ≥ 25%), luminal B HER2+ (ER+ and/or PR+, HER2/neu+), HER2/neu non-luminal (ER/PR−, HER2/neu+) and TNBC (ER/PR−, HER2/neu−) [23].

### 2.8. Immunohistochemistry

CXCR4 immunohistochemistry was performed at the National Health Laboratory Service (NHLS) Laboratory at Inkosi Albert Luthuli Central Hospital. CXCR4 staining was categorized according to the intensity and percentage of stained cells. Overall staining intensity for CXCR4 was scored into three categories as follows: 0 (absence of staining), 1 (mild), 2 (moderate) and 3 (strongly positive). The proportion of stained cells was scored into four categories as follows: 1 (<10% positive cells) (low), 2 (10–50% positive cells), 3 (>50–80% positive cells) and 4 (>80% positive cells) [24]. The two scores were multiplied to obtain the immune reactivity (IRS) score. IRS scores were correlated with ^68^Ga-Pentixafor and ^18^F-FDG uptake.

### 2.9. Follow-Up

The patients were followed up with for a median of 17 months (range 4–48 months). The ^18^F-FDG and ^68^Ga-Pentixafor PET metrics were correlated with survival, and optimal cut-off points for predicting survival were computed. Treatment response was correlated with ^68^Ga-Pentixafor and ^18^F-FDG PET metrics as well as CXCR4 IHC staining.

### 2.10. Statistical Analysis

Using GPower software 3.1.9.7 for sample calculation, it was estimated that the sample size of 50 would allow for the detection of a minimum effect size of 0.44 with 80% power. The statistical data analysis was conducted in R Statistical computing software of the R Core Team, 2020, version 3.6.3. Multidimensional numerical variables were presented as correlation plots and the associations were assessed using correlation tests. Depending on the distribution of the numerical variables between two independent groups, mean or median differences were assessed using either a *t*-test or Wilcoxon test, respectively. All the inferential statistical analysis tests were conducted at 5% levels of significance. To evaluate the degree of correlation between SUVmax and PR status, ER status and Ki67 proliferation index as well as other non-normally distributed numerical variables, we performed the Spearman rank test. To assess for differences in PET metrics such as MTV, SUVmax, TLU and TLG across different molecular subtypes, we used the Kruskal–Wallis test. A Kaplan–Meier analysis was utilized to establish whether there was an association between HIV infection and PET/CT metrics (e.g., SUVmax) with survival outcomes. Receiver-operating characteristic (ROC) curve analysis was used to determine the optimal cut-off for PET/CT metrics that best predicted poor survival.

## 3. Results

### 3.1. Patient Clinical Characteristics

A total of 51 patients were prospectively evaluated with ^68^Ga-Pentixafor PET/CT, and the mean age was 53.7 (±10.7) years. A total of 47 out of 51 patients were primarily diagnosed with breast carcinoma without a history of previous malignant breast lesions or therapy. Five patients had recurrence (patient characteristics are summarized in Table 1). Five (10%) had luminal A, nineteen (40%) had luminal B, five (10%) had HER2-enriched, twenty-one (40%) had triple-negative and one patient had an atypical molecular subtype of breast cancer. Overall, 12%, 53% and 35% of the patients had local, locally advanced and metastatic disease, respectively. None of the patients had undergone surgery at the time of the PET/CT scans. Seven of the patients were on hormonal therapy at the time of the scan, and one patient had undergone chemotherapy.

Nineteen patients (37%) were HIV-positive and 33 (63%) were HIV-negative. HIV-positive patients with breast cancer were significantly younger than HIV-negative patients, with median ages of 49.0 (43.0–56.5) and 53.0 (46.0–72.0), respectively (*p* = 0.054). The ^68^Ga-Pentixafor TLU was significantly higher in HIV-positive compared to HIV-negative breast cancer patients (median (Q1–Q3) 376 (219–881) and 174 (105–557), respectively. *p* = 0.038 (Table 2)). The ^68^Ga-Pentixafor MTV was significantly higher in HIV-positive compared to HIV-negative patients (median (Q1–Q3) 376 (219–881) and 174 (105–557), respectively. *p* = 0.058). The Ki67 was higher in the HIV-positive patients compared to the HIV-negative patients, and seven (41.2%) of the HIV-positive patients had Ki67 > 70% versus five (15.2%) of the HIV-negative; however, this did not reach statistical significance (*p* = 0.077).

### 3.2. Correlation Between ^68^Ga-Pentixafor PET/CT Metrics and Prognostic Factors

**Figure 2:**
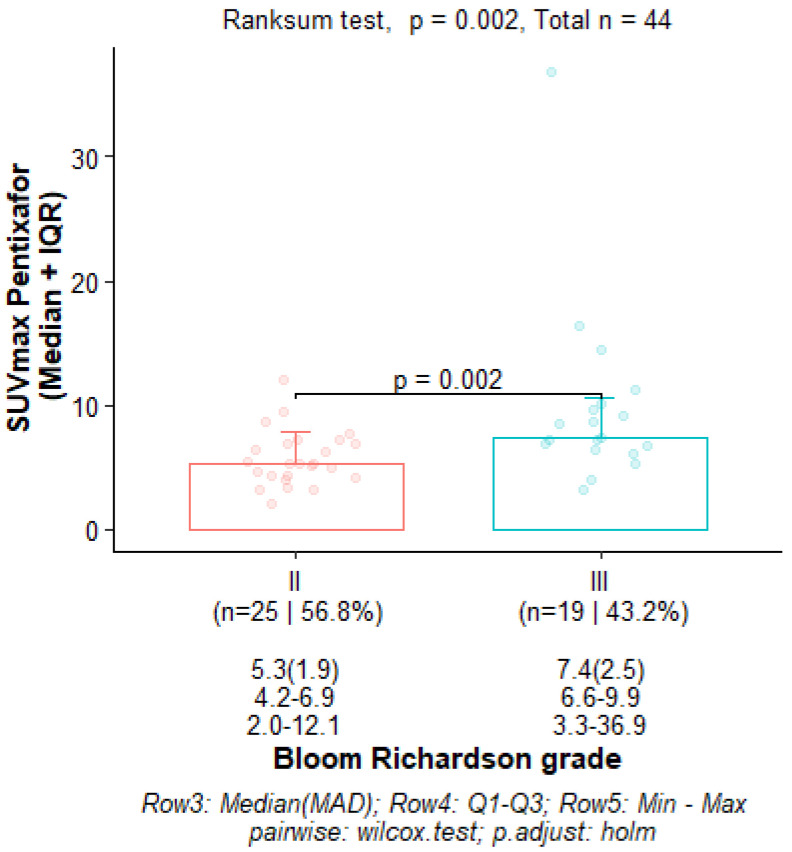
Box and whisker plot showing a higher SUVmax Pentixafor in Bloom Richardson grade III compared to grade II.

**Figure 3:**
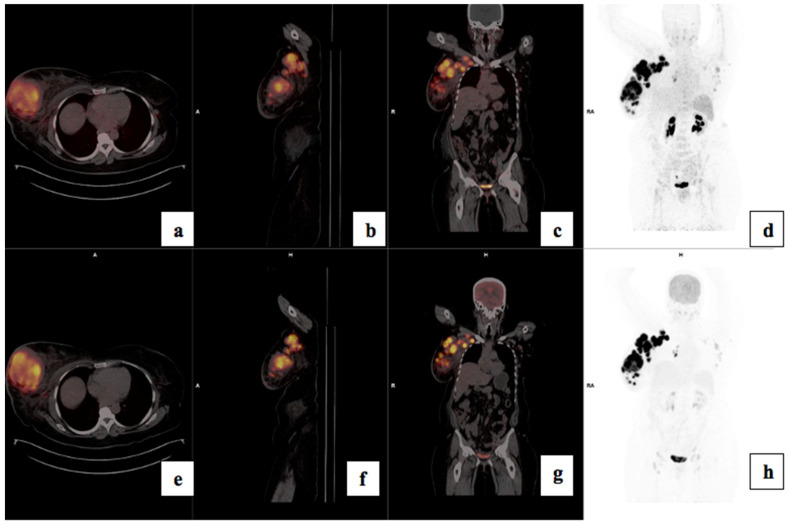
Shows ^68^Ga-Pentixafor (**a**–**d**) and FDG (**e**–**h**) images of a 56-year-old with TNBC T4bN2M0, Bloom Richardson Grade III Ki67 50%, showing intense uptake on both tracers in the right breast (**a**,**e**) and axillary lmph nodes (**b**,**f**). SUVmax was 36.85 on ^68^Ga-Pentixafor and 77.94 on ^18^F-FDG. The TBRs for FDG and Pentixafor were 19.5 and 17.4 in the primary and 7.6 and 7.2, respectively, in the axillary lymph nodes. The patient also has HIV with reactive inguinal lymph nodes, demonstrating mild uptake on both tracers (**d**,**h**).

There was a statistically significant correlation between tumor grade and CXCR4 expression: the median (Q1–Q3) ^68^Ga-Pentixafor PET/CT SUVmax was 5.32 (4.24–6.96) for Bloom Richardson grade II and 7.40 (6.77–9.57) for grade III (*p* = 0.002) as shown graphically in Figure 2. The SUVmean for Pentixafor was significantly higher in patients with a Ki67 > 70% median (Q1–Q3) SUVmean of 4.12 (4.00–5.78) compared to 3.45 (2.82–3.99) in patients with Ki67 < 70% (*p* = 0.008). Also, the ^68^Ga-Pentixafor SUVmean was significantly higher in patients with Bloom Richardson grade III tumors median (IQR) 4.23 (3.59–5.27) compared to grade II 3.31 (2.75–3.79) (*p* = 0.004). Figure 3 shows an example of an HIV-positive breast cancer patient with Bloom Richardson grade III triple-negative breast cancer showing intense uptake on both tracers.

The median SUVmax was higher in TNBC compared to the luminal subtypes: median SUVmax 7.10 (5.95–9.63) for TNBC, 5.24 (4.26–7.18) for luminal A, 5.45 (4.30–7.09) for luminal B and 5.10 (4.18–7.40) for HER2-enriched.

There was no statistically significant correlation between the ^68^Ga-Pentixafor SUVmax of the primary and molecular subtypes: the median SUVmax was 7 for ER-positive disease (*n* = 24) and 5.4 for ER-negative disease (*n* = 25); 6.5 for PR-positive disease (*n* = 31) and 5.4 for PR-negative disease (*n* = 18); 6.4 for HER2-negative and 5.2 for HER2-positive disease. The median SUVmax was slightly lower for HIV-negative patients (6.07 (4.22–7.49)) compared to (6.99 (5.46–8.68)) for HIV-positive patients. There was no statistically significant correlation between ^68^Ga-Pentixafor SUVmax and the T, N or M stages of disease.

### 3.3. Comparison of FDG and Pentixafor

^68^Ga-Pentixafor detected 49/51 (96%) primary breast lesions and ^18^F-FDG detected 40/40 (100%) lesions in the patients that had both scans (Table 3). In a lesion-based analysis, of the 40 patients that underwent both scans, ^18^F-FDG detected 284 lesions and ^68^Ga-Pentixafor detected 257 (90%) lesions. ^68^Ga-Pentixafor detected 127/135 (94%) axillary lymph node metastasis, 8/10 (80%) extra-axillary lymph nodes, 13/15 (87%) liver metastasis, 24/24 (100%) lung metastasis, 36/66 (54%) bone metastases and 3/3 brain metastases. Also, ^18^F-FDG detected 1/3 (33%) brain metastases and one metastasis in the skull was missed by ^18^F-FDG.

**Figure 4:**
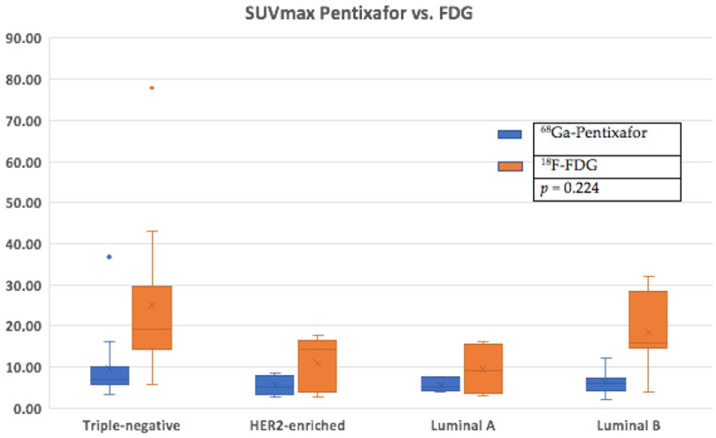
A higher median SUVmax for ^18^F-FDG (orange) is shown compared to ^68^Ga-Pentixafor (blue) across the different histological subtypes.

**Figure 5:**
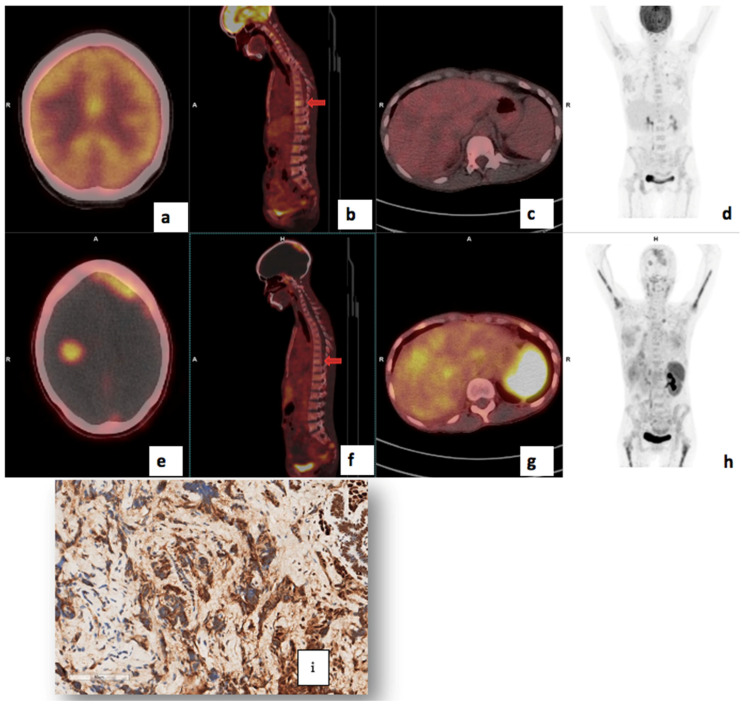
A 41-year-old with bilateral T4bN2M0 luminal B breast cancer, Bloom Richardson Grade II, HIV-positive, Ki67 30%, and metastasis to the lungs, bone, liver (**c**,**g**) and brain (**a**,**e**). Using ^68^Ga-Pentixafor, mild uptake was observed in the breast primary, SUVmax 4.6 (**d**,**h**). The brain metastasis were more clearly visualised on ^68^Ga-Pentixafor imaging (**e**) compared to ^18^F-FDG (**a**) due to the absence of physiological brain uptake with Pentixafor. The liver metastasis are also detected on Pentixafor (**g**) and not seen on FDG (**c**). However, the skeletal metastasis are seen on FDG (**b**) and not Pentixafor (**f**) likely due to higher physiological bone marrow uptake on Pentixafor (**h**). CXCR4 IHC showed strongly positive staining in 60% of the tumour cells (**i**).

**Figure 6:**
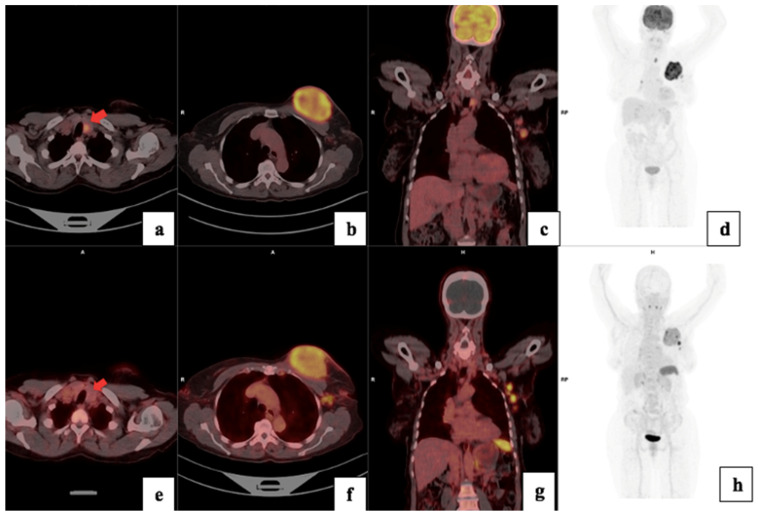
^18^F-FDG (**a**–**d**) and ^68^Ga-Pentixafor (**e**–**h**) images showing increased uptake in the left breast primary (**b**,**f**) and left axillary lymph nodes (**c**,**g**) in a a 64-year-old female with T3N1M0 triple-negative invasive ductal carcinoma of the left breast. The primary tumour exhibited a Ki67 index of 90% and an SUVmax of 7.26, on ^68^Ga-Pentixafor and 20.79 on ^18^F-FDG. Notably, ^18^F-FDG showed high uptake in a benign thyroid lesion (**a**), which was negative using Pentixafor as indicated by the arrows (**e**).

There was a higher tracer uptake on ^18^F-FDG PET/CT compared to ^68^Ga-Pentixafor, as shown in Figure 4. The median ^68^Ga-Pentixafor PET/CT SUVmax was 6.35 (4.62–7.64) and 16.9 (12.5–24.6) for ^18^F-FDG. The median total lesion uptake (TLU) was 242 (115–647) for ^68^Ga-Pentixafor and 416 (203–1120) for ^18^F-FDG (Table 4). The median MTV was 89 (28–175) for ^68^Ga-Pentixafor and 54 (27.1–1560) for ^18^F-FDG (Figure 5). In two patients, ^68^Ga-Pentixafor detected brain metastases that were missed by ^18^F-FDG PET/CT, and in one patient, ^68^Ga-Pentixafor detected a skull lesion that was overlooked by ^18^F-FDG. In one patient, ^18^F-FDG detected skeletal metastases in the spine that were not seen by ^68^Ga-Pentixafor. ^18^F-FDG also detected one false-positive benign thyroid lesion that was negative using ^68^Ga-Pentixafor (Figure 6).

### 3.4. Visual Analysis

Forty-nine (96%) of the fifty-one primary breast lesions were visually detectable by ^68^Ga-Pentixafor PET/CT with a mean SUVmax of 7.36 ± 5.4 *p* < 0.001. A SUVmax of four or more correlated with visually detectable disease for ^68^Ga-Pentixafor.

### 3.5. Treatment Response

Fifteen (29%) of the fifty-one patients had a complete response to therapy, eleven (22%) had disease progression, fourteen (27%) had a partial response, eleven (22%) had stable disease and one patient died before the initiation of treatment. There was no statistically significant correlation between PET/CT metrics and treatment response.

### 3.6. Survival Analysis

**Figure 7:**
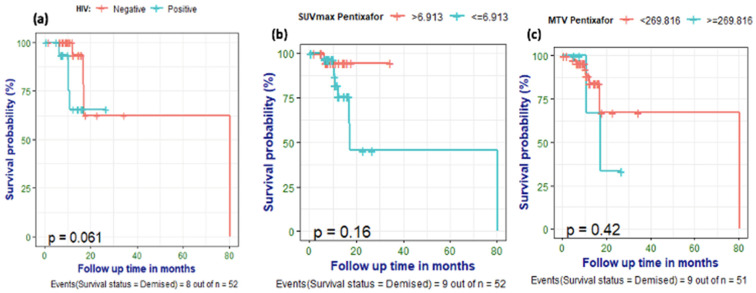
Kaplan–Meier curves showing a trend towards poorer survival in HIV-positive breast cancer patients (**a**) and no significant correlation between survival and Pentixafor SUVmax (**b**) and MTV Pentixafor (**c**).

Nine (17%) of the patients had died by the time of data analysis. ^18^F-FDG and ^68^Ga-Pentixafor PET metrics were correlated with survival, and optimal cut-off points for predicting survival were computed. A SUVmean of ≥3.291 was predictive of poor survival with a sensitivity of 71%, specificity of 63% and AUC of 0.662. SUVmean had the highest AUC, indicating better predictive performance compared to other PET metrics. A primary ^68^Ga-Pentixafor MTV of ≥270 cm^3^ correlated with poor survival with an accuracy of 83%, specificity of 92% and moderate AUC (0.631) (Table 5). The optimal cut-off for poorer survival for the TBR (breast) was ≤6.32 with a sensitivity of 100. Patients with elevated ALP were significantly more likely to succumb to the disease than patients with normal ALP (*p* = 0.044). There was an indication that HIV-negative patients might have a longer survival than HIV-positive breast cancer patients, although this difference did not reach statistical significance (*p* = 0.061). Furthermore, patients with a ^68^Ga-Pentixafor SUVmax of ≥6.9 showed a trend toward shorter survival, as demonstrated on the Kaplan–Meier curves (Figure 7), even though statistical significance was not reached (*p* = 0.18).

### 3.7. Correlation of PET/CT Metrics with CXCR4 Immunohistochemistry Staining

**Figure 8:**
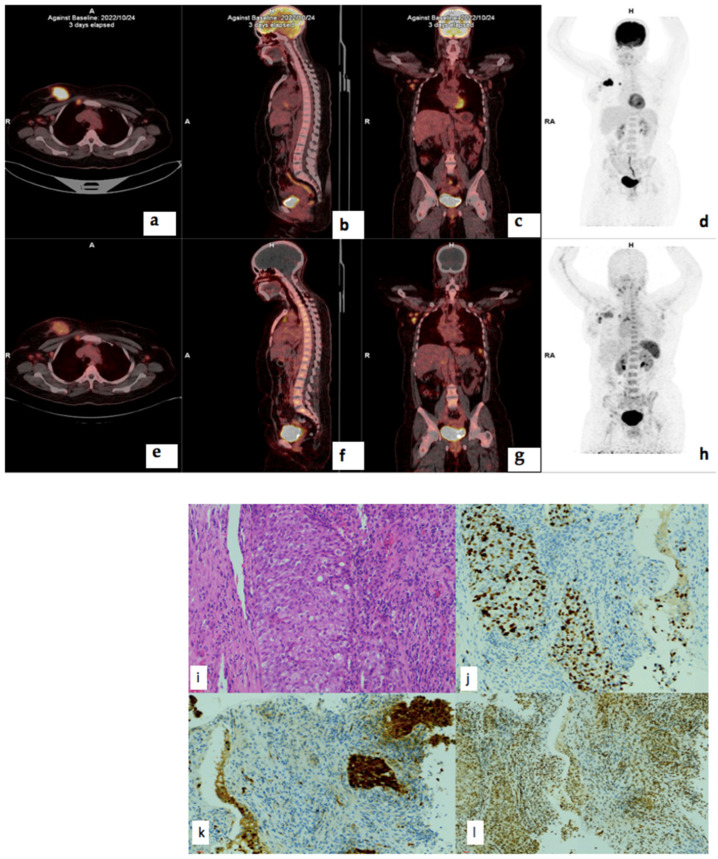
^18^F-FDG (**a**–**d**) and ^68^Ga-Pentixafor (**e**–**h**) PET/CT images of a 41-year-old female with right breast cancer T3N1M0, triple-positive, luminal B molecular subtype, with a Ki67 index of 70% showing moderate Pentixafor uptake in the breast primary (**e**), right internal mammary and axillary lymph nodes (**g**). Corresponding H&E (**i**), p16 (**j**), Ki67 (**k**), CXCR4 (**l**) IHC staining slides of the primary tumur showed moderate (++) staining in 90% of the tumor cells.

There was no statistically significant correlation between ^68^Ga-Pentixafor or ^18^F-FDG PET and CXCR4 IHC staining intensity, % stained cells or IRS. The correlation coefficient for ^18^F-FDG MTV and CXCR4 IRS was −0.279 (*p* = 0.222) and 0.192 (*p* = 0.405) for ^68^Ga-Pentixafor. There was also no correlation between CXCR4 IRS and survival *r* = −0.38 (*p* = 0.090). There was no correlation between CXCR4 IRS and ^68^Ga-Pentixafor SUVmax correlation coefficient was -0.035 (*p* = 0.879) and, for ^18^F-FDG, *r* = 0.024 (*p* = 0.919). Figure 8 shows histopathology slides of a strongly positive CXCR4 IHC staining in a 40 year old female with triple positive breast cancer.

## 4. Discussion

In this prospective study, comparing ^68^Ga-Pentixafor with ^18^F-FDG PET, we have shown that in vivo imaging of CXCR4 using the ^68^Ga-Pentixafor is possible for both the primary lesion and metastasis; however, ^68^Ga-Pentixafor is inferior to ^18^F-FDG PET with a sensitivity of 96% versus 100% for ^18^F-FDG. While the stage of disease did not change between the two scans, the intensity of ^68^Ga-Pentixafor accumulation was significantly lower compared to ^18^F-FDG, confirming the findings by Vag et al. that ^68^Ga-Pentixafor cannot replace ^18^F-FDG PET in the staging of breast cancer [12].

**Figure 9:**
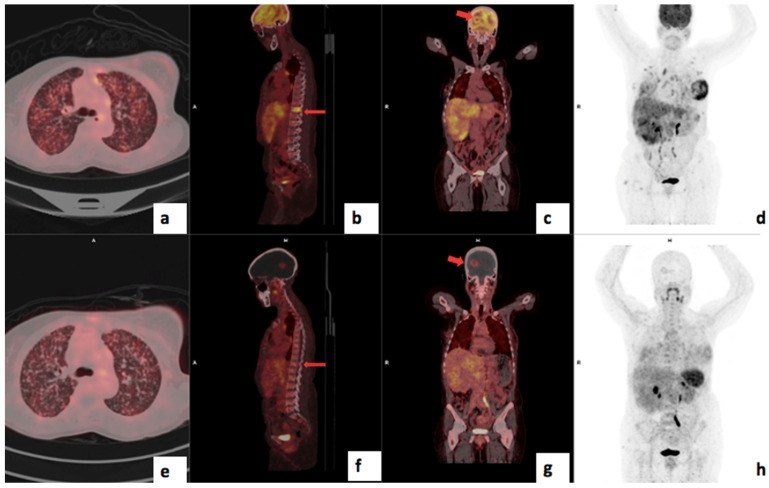
A 48-year-old female with left T3N1M1 breast cancer showing increased heterogeneous uptake in the left breast primary (SUVmax 13.1), with brain, liver, lung and skeletal metastasis using ^18^F-FDG PET/CT (**a**–**d**). The lesions are seen on ^68^Ga-Pentixafor (**e**–**h**) but appear less intense (SUVmax 4.26). The brain lesion is more visible using Pentixafor (**g**) due to lower brain uptake than FDG (**c**) and the vertebral lesion is more visible on FDG (**b**) than Pentixafor (**f**) due to higher physiological marrow uptake (**h**).

In addition, because of the less intense physiological uptake of ^68^Ga-Pentixafor in the brain, this novel tracer allows for better visualization of metastasis in the skull and brain, as demonstrated in Figure 6. This is in agreement with findings by Buck et al. [25]. As the brain is an obligate user of glucose, there is a high physiological uptake of FDG, which may obscure both intracranial and skull lesions. However, ^68^Ga-Pentixafor, similar to [^18^F]-FDG, also accumulated in reactive lymph nodes, and therefore, the biopsy of suspicious lymph nodes remains invaluable for confirmation of metastatic spread, especially in areas outside the normal/expected lymphatic drainage of the breast. ^68^Ga-Pentixafor missed skeletal metastasis in the spine in two patients likely due to the high physiological bone marrow accumulation of this tracer; an example is shown in Figure 9. The high but variable physiological uptake on ^68^Ga-Pentixafor is due to CXCR4 expression on hematopoietic stem cells and progenitor cells in the bone marrow [26]. Further, it is possible that the high bone marrow accumulation of ^68^Ga-Pentixafor is attributed to an immune response triggered by stress signals originating from peripheral tissues, which subsequently activate the bone marrow [26]. Solid tumors, including breast cancer, induce a state of chronic inflammation that causes myelopoiesis in the bone marrow.

Disease progression in breast cancer is intricately dependent on the tumor microenvironment (TME), a complex network of extracellular matrix (ECM) proteins, immune cells and non-cancerous stromal cells, such as endothelial cells, adipocytes, and fibroblasts [27]. Cancer-associated fibroblasts (CAFs) secrete transforming growth factor (TGF) β which can neutralize the anti-tumor response of NK cells, neutrophils and macrophages, thus enhancing the ability of the cancer to evade the immune system [28]. TGFβ1 upregulates CXCR4 expression in breast cancer cells and plays a prominent role in metastasis by enhancing angiogenesis and suppressing immune surveillance at secondary metastatic sites [29,30].

**Figure 10:**
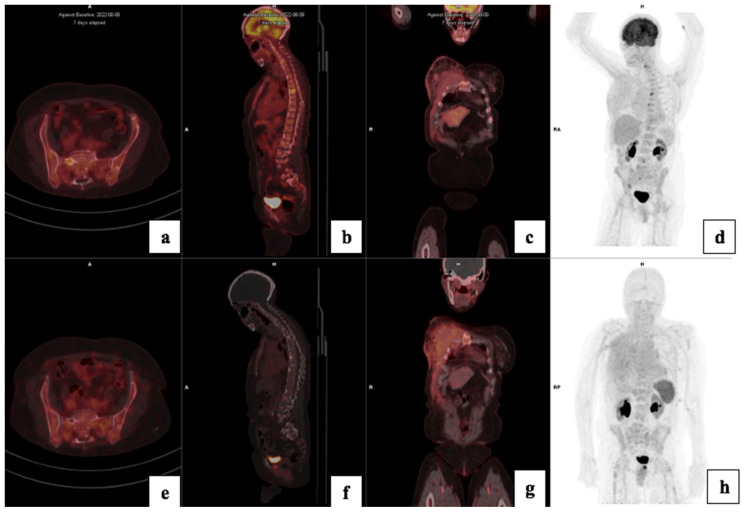
A 73-year-old with luminal A T4cN2M0 Bloom Richardson Grade III right breast cancer, demonstrating very mild uptake in the right breast and overlying soft tissue: SUVmax 2.9 using ^18^F-FDG (**c**,**d**) and 4.04 using ^68^Ga-Pentixafor (**g**,**h**). Notably, FDG detected skeletal metastases (**a**,**b**) that were missed using ^68^Ga-Pentixafor (**e**,**f**). CXCR4 expression was mild in 15% of the tumour cells on IHC staining.

Interestingly, ^68^Ga-Pentixafor SUVmax and SUVmean correlated positively with the Bloom Richardson tumor grade and tumor proliferation index Ki67 (Figure 10). Although this finding is conflicting with findings by Vag et al., where there was no correlation [12], it is expected that more aggressive tumors, including high-grade tumors with a high proliferation index, will have higher expression of CXCR4 [31,32]. The conflicting results could be due to a small sample size of 18 patients in that study vs. 51 patients in this current study and the higher prevalence of HIV and more aggressive variants of breast cancer in our cohort. However, similar to the existing literature, there was no statistically significant correlation between SUVmax and molecular subtypes of breast cancer.

The ^68^Ga-Pentixafor SUVmax for TNBC was higher than the luminal subtypes in this current study; however, this did not reach statistical significance due to the modest sample size. The median ^68^Ga-Pentixafor PET/CT SUVmax values in patients with luminal A, luminal B, HER2-enriched and triple-negative disease were 5.24 (4.26–7.18), 5.45 (4.30–7.09), 5.10 (4.18–7.40) and 7.10 (5.95–9.63), respectively (*p* = 0.224). Figure 9 shows an example of a patient with luminal A molecular subtype breast cancer with very low avidity in the breast primary and skeletal disease using ^68^Ga-Pentixafor; the lesions are clearly visible using ^18^F-FDG. This is in agreement with a study by Vazquez et al. who demonstrated a higher CXCR4 expression in triple-negative samples compared to in luminals A and B and HER2-enriched in the molecular subtype analysis of breast cancer tissue RNA sequencing data from the Cancer Genome Atlas (TCGA) breast cancer cohort [33].

HIV infection was associated with higher ^68^Ga-Pentixafor tracer accumulation. The median TLU was 174 (105–557) for HIV-negative patients and 376 (219–881) for HIV-positive patients (*p* = 0.038). CXCR4 is a co-receptor for the T-trophic HIV seen in the late phase of HIV infection associated with a more rapid deterioration of the immune system and faster progression to AIDS [34]. While early studies showed the risk of breast cancer in HIV-infected individuals to be lower than in the general population, recent studies have demonstrated a more aggressive clinical course, poorer outcome and younger age at diagnosis of breast cancer in HIV-infected individuals compared to the general population [35]. This was confirmed in this current study, where HIV-infected breast cancer patients were significantly younger (median age 49.0 (43.0–56.5) and 53.0 (46.0–72.0), respectively; *p* = 0.054) and had a higher Ki67 proliferative index. This means that HIV-infected patients may need a more aggressive treatment approach than their HIV-uninfected counterparts.

In HIV-1 infection, CXCR4 predominantly manifests at the advanced stages, correlating with rapid CD4^+^ T cell depletion and disease progression [36]. Recent evidence suggests that CXCR4 antagonists slow down disease progression and potentially delay the onset of AIDs. Given the dual role of CXCR4 in HIV pathogenesis and cancer biology, the therapeutic application of CXCR4 antagonists in HIV-positive breast cancer patients may have a potentially synergistic effect: slowing down the progression of HIV while simultaneously impeding tumor proliferation, chemotaxis and metastatic dissemination of breast cancer. Future studies should explore the potential synergistic effect of CXCR4 antagonists in cancer patients with HIV infection and the role of CXCR4-targeted imaging in selecting patients who will potentially benefit from this therapy.

CXCR4 has been established as an independent prognostic biomarker across multiple malignancies, including breast cancer, where its expression is strongly associated with aggressive tumor behavior, metastatic potential and poor clinical outcomes [37]. In this study, we investigated the utility of ^68^Ga-Pentixafor uptake as a prognostic tool in primary tumors. Our findings demonstrated that ^68^Ga-Pentixafor MTV ≥ 270 cm^3^ was significantly associated with worse survival outcomes with an accuracy of 83% and specificity of 92%. A SUVmean of ≥3.291 was predictive of poor survival with a sensitivity of 71%, specificity of 63% and AUC of 0.662; SUVmean had the highest AUC, indicating better predictive performance compared to other PET metrics, which suggests that this parameter has potential as a robust prognostic biomarker.

CXCR4 is an appealing target for diagnostic and therapeutic approaches in cancer patients and has shown promise in hematological malignancies [38,39]. It has been demonstrated that over 75% of TNBC express CXCR4, which could potentially be targeted, particularly in patients who are refractory to chemotherapy [40]. CXCR4 antagonists disrupt the CXCR4/CXCL12 signaling pathway, thereby enhancing the efficacy of chemotherapy by increasing drug delivery to the tumor and reducing resistance mechanisms driven by hypoxia and stromal interactions [41]. Additionally, by remodeling the immunosuppressive TME, CXCR4 antagonists improve the efficacy of immune checkpoint inhibitors [42], improve immune infiltration into tumors and reprogram immune suppression [43]. The synergistic effect underscores the potential of CXCR4-targeted therapy in addressing immune evasion inherent to TNBC and the importance of studying gene expression profiles to identify biomarkers that can be targeted for personalized combination therapies.

A few CXCR4 antagonists have been investigated in TNBC models. CXCR4 antagonists target CXCR4-upregulated tumor cells, block the CXCR4 receptor from binding to its ligand SDF-1 and inhibit bone and lung metastasis [44,45]. Several studies have demonstrated that CXCR4 antagonists also increase chemosensitivity and enhance the antitumor efficacy of chemotherapy, including docetaxel [46,47].

Plerixafor (AMD3100) is a specific CXCR4 inhibitor approved by the Food and Drug Administration (FDA, USA) as a CXCR4-targeted therapy for hematopoietic stem cell mobilization in hematological malignancies [13]. It has been shown that the addition of Plerixafor to the existing chemotherapies, radiotherapies or immunotherapies enhances their therapeutic efficacy in various cancers, including breast cancer [35]. Preclinical studies show that CTCE-9908, another CXCR4 inhibitor, competitively inhibits the interaction between the CXCR4 receptor and its ligand SDF-1α, which blocks the function of CXCR4, thus suppressing tumor growth and metastasis [46]. Thus, CXCR4 inhibitors have the potential for the treatment of breast cancer, particularly TNBC, as they have a more avid concentration of ^68^Ga-Pentixafor. In a preclinical study, Bao et al. demonstrated a synergistic effect of a combination of CXCR4 antagonists (AMD3100) and radioligand therapies targeting cancer-associated fibroblasts (CAFs)- ^177^Lu-DOTAGA.(SA.FAPi)_2_ in tumor models of TNBC. The authors demonstrated a significant suppression of cell proliferation, migration and colony formation in cell cultures compared to monotherapy [40].

While CXCR4-targeted radionuclide therapies such as ^177^Lu/^90^Y Pentixather have shown promise in hematological malignancies [37,48], bone marrow toxicity remains a limitation in solid tumors due to high radiotracer uptake. Therefore, other radiotracers targeting CXCR4 with less bone marrow accumulation are being explored [49,50]. Also, non-radiolabeled “cold” CXCR4 antagonists such as Plerixafor may have a role in decreasing tumor proliferation in patients demonstrating CXCR4 expression in PET/CT imaging [51,52]. Therefore, future studies should establish the role of ^68^Ga-Pentixafor in selecting candidates for therapies targeting CXCR4.

In this current study, we found no correlation between the in vitro CXCR4 expression of IHC and ^68^Ga-Pentixafor uptake; this is similar to the study by Vag et al. [12]. The discrepancy between the in vivo expression demonstrated in imaging and the findings on immunohistochemistry is because CXCR4 IHC detects both membranous and cytoplastic CXCR4, while ^68^Ga-Pentixafor is unable to pass the membrane and thus only detects membrane-localized receptor expression [53]. Also, a discrepancy has been reported between diffuse cytoplasmatic CXCR4 in 81% compared to membrane-localized CXCR4 in only 25% of breast tumors using immunohistochemistry staining [54,55]. Therefore, because cytoplasmatic CXCR4 does not contribute to signal intensity in PET imaging, CXCR4-targeted PET imaging has a lower overall signal intensity of breast cancer cells than possibly assumed by in vitro examinations [12].

This study is limited by the unavailability of ^18^F-FDG PET/CT correlation in 10 of the 51 patients. Six (10%) of the study patients were excluded from the final analysis as they had suboptimal scans, and this led to a relatively small sample size with a heterogeneous group of molecular subtypes, which may be a source of bias. In addition, patients were followed up for a median of 17 months (range: 4 to 48 months) after recruitment; therefore, some patients were followed up for a shorter time. A standardized or longer follow-up time would have allowed for a better assessment of the impact of CXCR4 expression on survival. Lastly, confirmation with histopathological biopsy could not be performed in all the metastatic lesions; therefore, conventional imaging (CT and ultrasound) together with clinical follow-up were used to differentiate between benign and malignant metastatic lesions. The unenhanced CT was not diagnostic quality, which may limit its sensitivity for detecting small lesions.

## 5. Conclusions

In conclusion, ^68^Ga-Pentixafor had a sensitivity of 96% and a specificity of 100% for detecting primary breast cancer. In addition, ^68^Ga-Pentixafor exhibited a significantly higher uptake in patients with higher tumor grade, high proliferative index and triple-negative breast cancer (TNBC), as well as HIV-infected breast cancer patients, highlighting the potential clinical utility and prognostic role of CXCR4-targeted PET imaging in aggressive breast cancer. Notably, ^68^Ga-Pentixafor complements ^18^F-FDG by detecting more metastasis in the brain and the skull where FDG has limitations, while ^18^F-FDG remains superior for detecting skeletal metastasis. ^68^Ga-Pentixafor uptake correlated significantly with HIV infection, underscoring the need to explore the potential synergistic effect of CXCR4 antagonists in HIV-infected breast cancer patients. ^68^Ga-Pentixafor SUV mean ≥ 3.291 and MTV ≥ 270 cm^3^ were associated with poor survival. Future research should further explore the potential of CXCR4-targeted PET imaging in selecting patients with triple-negative breast cancer and high-grade breast cancer who may benefit from CXCR4-targeted therapies and monitoring response to therapy, particularly in the context of HIV co-infection.

## Figures and Tables

**Table 1:** Summary of the demographic and clinical details of the study population.

Clinical Details	
Age median (IQR)	51 (36–81)
Ki67	50 (10–90%)
**T stage**	N = 51 (%)
1	1 (2.0%)
2	5 (10.0%)
3	12 (24.0%)
4	33 (64.0%)
**N stage**	
N1	26 (52%)
N2	19 (38%)
**Metastasis**	
Internal mammary	1
Bone	12
Liver	6
Lung	8
Brain	2
**Bloom Richardson grade**	
II	24 (55%)
III	19 (45%)
**Histology**	
IDC	45 (90%)
IDC (mucinous component)	2 (4%)
ILC	1 (2%)
Metaplastic	1 (2%)
**Molecular subtypes**	
Luminal A	5 (10%)
Luminal B	19 (40%)
HER2-enriched	5 (10%)
Triple-negative	21 (40%)
Atypical	1 (2%)
**Receptor expression**	
ER+/−	24/26
PR+/−	31/19
HER2+/−	40/9
**HIV infection**	
HIV-positive	18 (37%)
HIV-negative	33 (63%)
**Race**	
Black	36 (69%)
Indian	11 (21%)
White	3 (8%)
Mixed-race	1 (2%)

IDC = invasive ductal carcinoma; ILC = invasive lobular carcinoma; ER = estrogen receptor; PR = progesterone receptor; HER2 = human epidermal growth factor receptor 2.

**Table 2:** A significantly higher Pentixafor TLU was shown in HIV-positive breast cancer patients compared to HIV-negative patients (*p* = 0.038). TLU = Total lesion Uptake.

HIV	Negative (N = 33)	Positive (N = 18)	*p*-Value	Overall (N = 51)
TLU Pentixafor			Ranksum	
Median(Q1–Q3)	174 (105–557)	376 (219–881)	0.038	239 (112–677)
n(Min–Max)	32 (6.21–1150)	19 (60.7–5950)		51 (6.21–5950)

**Table 3:** Comparison of ^68^Ga-Pentixafor and ^18^F-FDG PET metrics for breast primary lesions.

	Median SUVmax	Median MTV	Median TLU	Median TBR (Liver)	Median TBR (Breast)	Sensitivity (%)	Specificity (%)
^68^Ga-Pentixafor	6.35	89	242	2.2	1.7	96	100
^18^F-FDG	16.9	54	416	9.8	4.8	100	100

TBR: tumor-to-background ratio; MTV: metabolic tumor volume; TLU: total lesion uptake.

**Table 4:** A statistically significant correlation was shown between FDG and Pentixafor PET metrics.

Variable 1	Variable 2	Correlation Coefficient	*p*-Value
^68^Ga-Pentixafor MTV	^18^F-FDG MTV FDG	0.523	<0.001
Ki67	^18^F-FDG SUVmean	0.362	0.022
CA 153	^68^Ga-Pentixafor TBR (liver)	0.589	0.034
^68^Ga-Pentixafor TLU	^18^F-MTV FDG	0.477	0.002
^68^Ga-Pentixafor TLU	^18^F-FDG TBR	0.418	0.002
^68^Ga-Pentixafor TLU	^18^F-FDG SUVmean	0.426	0.005
^68^Ga-Pentixafor TBR(breast)	^18^F-FDG SUVmax	0.667	<0.001
^68^Ga-Pentixafor TBR (breast)	^18^F-FDG TLG	0.583	<0.001
^68^Ga-Pentixafor TBR (breast)	^18^F-FDG SUVmean	0.553	<0.001
^68^Ga-Pentixafor TBR (breast)	^18^F-FDG TBR max	0.530	<0.001

TBR: tumor-to-background ratio.

**Table 5:** Correlation between Pentixafor PET metrics and survival.

Predictor	Direction	Optimal_Cutpoint	Youden	Accuracy	Sensitivity	Specificity	AUC	Prevalence
SUVmax Pentixafor	<=	6.913	0.286	0.490	0.857	0.429	0.592	0.143
SUVmean Pentixafor	<=	3.291	0.348	0.646	0.714	0.634	0.662	0.146
TLU_Pentixafor	>=	3676.708	−0.049	0.812	0.000	0.951	0.603	0.146
MTV_Pentixafor	>=	269.816	0.213	0.833	0.286	0.927	0.631	0.146
TBR_(liver)_ Pentixafor	<=	4.486	0.095	0.224	1.000	0.095	0.558	0.143
TBR_(breast)_ Pentixafor_	<=	6.342	0.048	0.184	1.000	0.048	0.507	0.143

MTV: metabolic tumor volume TBR: tumor-to-background ratio. AUC: area under the curve. TLU: total lesion uptake.

## Data Availability

The raw data supporting the conclusions of this article will be made available by the authors on request.

## Abbreviations

CXCR4	Chemokine receptor 4
FDG	Fluorodeoxyglucose
HIV	Human Immunodeficiency Virus
IHC	Immunohistochemistry
PET	Positron Emission Tomography
RT	Radiation therapy
SUV	Standardised Uptake Value
TBR	Tumor-to-breast background ratio
TNBC	Triple negative breast cancer
TLR	Tumor-to-liver background ratio
MTV	Metabolic tumor volume
TLU	Total lesion uptake
TLG	Total lesion glycolysis
